# Role of matrix metalloproteinases in multi-system inflammatory syndrome and acute COVID-19 in children

**DOI:** 10.3389/fmed.2022.1050804

**Published:** 2022-12-05

**Authors:** Nathella Pavan Kumar, Aishwarya Venkataraman, Poovazhagi Varadarjan, Arul Nancy, Anuradha Rajamanickam, Elilarasi Selladurai, Thangavelu Sankaralingam, Kannan Thiruvengadam, Ramya Selvam, Akshith Thimmaiah, Suresh Natarajan, Ganesh Ramaswamy, Sulochana Putlibai, Kalaimaran Sadasivam, Balasubramanian Sundaram, Syed Hissar, Uma Devi Ranganathan, Thomas B. Nutman, Subash Babu

**Affiliations:** ^1^ICMR – National Institute for Research in Tuberculosis, Chennai, India; ^2^Institute of Child Health and Hospital for Children, Chennai, India; ^3^National Institutes of Health-National Institute for Research in Tuberculosis – International Center for Excellence in Research, Chennai, India; ^4^Dr. Mehta’s Children’s Hospital, Chennai, India; ^5^Rainbow Children’s Hospital, Chennai, India; ^6^Kanchi Kamakoti CHILDS Trust Hospital, Chennai, India; ^7^Laboratory of Parasitic Diseases, National Institute of Allergy and Infectious Diseases, National Institutes of Health, Bethesda, MD, United States

**Keywords:** MIS-C, COVID-19, seropositive, MMPs, biomarker

## Abstract

**Introduction:**

Multisystem Inflammatory Syndrome in children (MIS-C) is a serious inflammatory sequela of SARS-CoV2 infection. The pathogenesis of MIS-C is vague and matrix metalloproteinases (MMPs) may have an important role. Matrix metalloproteinases (MMPs) are known drivers of lung pathology in many diseases.

**Methods:**

To elucidate the role of MMPs in pathogenesis of pediatric COVID-19, we examined their plasma levels in MIS-C and acute COVID-19 children and compared them to convalescent COVID-19 and children with other common tropical diseases (with overlapping clinical manifestations).

**Results:**

Children with MIS-C had elevated levels of MMPs (*P* < 0.005 statistically significant) in comparison to acute COVID-19, other tropical diseases (Dengue fever, typhoid fever, and scrub typhus fever) and convalescent COVID-19 children. PCA and ROC analysis (sensitivity 84–100% and specificity 80–100%) showed that MMP-8, 12, 13 could help distinguish MIS-C from acute COVID-19 and other tropical diseases with high sensitivity and specificity. Among MIS-C children, elevated levels of MMPs were seen in children requiring intensive care unit admission as compared to children not needing intensive care. Similar findings were noted when children with severe/moderate COVID-19 were compared to children with mild COVID-19. Finally, MMP levels exhibited significant correlation with laboratory parameters, including lymphocyte counts, CRP, D-dimer, Ferritin and Sodium levels.

**Discussion:**

Our findings suggest that MMPs play a pivotal role in the pathogenesis of MIS-C and COVID-19 in children and may help distinguish MIS-C from other conditions with overlapping clinical presentation.

## Introduction

Children mostly have either mild or no symptoms of SARS-CoV2 infection but may rarely manifest with an inflammatory condition 1–2 months after acute infection, now classified as multisystem inflammatory syndrome in children (MIS-C) (1–4). Symptoms of MIS-C such as fever, multi-organ dysfunction and elevated inflammatory markers are largely similar to acute COVID-19 of adults or Kawasaki disease in children (5, 6). However, MIS-C has distinct immunological features that are different from adult COVID-19 or Kawasaki disease with respect to cytokine profiles and immune cell compartments (7–9). Although we are yet to fully understand the pathogenesis of MIS-C, the presence of auto-antibodies including those directed at the casein kinase family of proteins have been reported (8, 9). The exact pathogenesis of MIS-C remains vague, with virus-induced post-infective immune dysregulation appearing to play a leading role. Previously published data has reported that overall MIS-C prognosis is good and reported mortality rates are 0–4% (10).

Matrix metalloproteinases (MMPs) are a family of zinc-dependent extracellular matrix remodeling enzymes that have the capacity to degrade almost every component of the extracellular matrix (11). Alterations in MMP levels can lead abnormal degradation of the extracellular matrix and result in pathology in most tissues (12). MMPs are implicated in all lung pathologies (13) and also involved in inflammation, modulating the synthesis and the release of cytokines and chemokines, and in cell growth, proliferation, and remodeling (14). Hence, we hypothesized that plasma levels of MMPs would be reflective of pathology in MIS-C. Studies from animal models have shown that an increase in MMP8, MMP9, and MMP14 levels in the lungs post SARS-CoV-2 infection was associated with degradation of lung extra-cellular matrix components, suggesting that MMP proteolytic activity in SARS-CoV-2 infection may be a potential target for COVID-19 treatment (15). Subsequently another study also reported that improper expression of several MMPs was correlated to lung disease of SARS-CoV-2 infection and the main findings from this study also reveals that MMPs are emerging as an important component of COVID-19 immunopathogenesis (16). Moreover, MMPs could potentially serve as biomarkers of MIS-C and enable its discrimination from acute COVID-19 as well as multiple other inflammatory and/or infectious conditions in children. To this end, we studied the association of a large panel of circulating MMPs in MIS-C, acute COVID-19 and children with other diseases in well-defined clinical cohort. We report that MIS-C and acute COVID-19 are characterized by heightened levels of MMPs (which also reflects disease severity) and that certain MMPs can help distinguish MIS-C from acute COVID-19 and other diseases.

## Materials and methods

### Ethics statement

Informed consent was obtained from parent/guardians of all children along with assent where appropriate. The Internal Ethics Committee (IEC) of the participating institutes approved the study. The study was also registered at Clinical Trials Registry India (CTRI/2021/01/030605). The study was also registered with Clinical Trials registry clinicaltrials.gov (No: NCT04844242).

### Study population and procedures

We prospectively enrolled children admitted to Kanchi Kamakoti CHILDS Trust Hospital (KKCTH), Chennai, India Institute of Child Health, Dr Mehta’s Children Hospital, Rainbow Children’s Hospital, from 1 June 2020 to 30 September 2020 with MIS-C, acute COVID-19, other diseases with include other infectious diseases (dengue, scrub typhus. Salmonella typhi infection [enteric fever]) or non-infectious diseases (e.g., systemic lupus erythematosus, diabetic ketoacidosis, Kawasaki’s disease), convalescent COVID-19 and control children. The study population and the enrolment criteria have been previously described (17). Briefly, we included children of either sex between 1 and 18 years of age who or whose parents were willing to provide informed consent/assent. Blood collection was performed prior to any immunomodulatory medication. Plasma was isolated and used for measuring multiple immune parameters. The demographic, epidemiological, medical and laboratory data have been previously reported (17) and are described in Tables 1, 2. COVID-19 disease severity was defined according to the Ministry of Health and Family Welfare (MOHFW) guidelines (18) issued by Government of India and children with MIS-C were diagnosed according to the World Health Organisation (WHO) case definition (19) and all the enrolled MIS-C cases have no other microbial or viral inflammatory focus. Children who were both SARS-CoV-2 RT-PCR negative and seronegative and who presented to the hospital for elective surgery were used as controls. They had no other co-morbid conditions and no history of contact with anyone with COVID-19. SARS-CoV-2 real-time reverse-transcriptase polymerase chain reaction (RT-PCR) was performed by Indian Council of Medical Research (ICMR) approved laboratories. The inclusion for convalescent COVID was determined by serology using the iFlash^®^ SARS-CoV-2 IgG chemiluminescence antibody assay (CLIA) (YHLO Biotechnology Corporation, Shenzhen, China) according to the manufacturer’s instructions. An IgG antibody titre of ≥ 10 AU/ml was considered positive (Table 1).

**TABLE 1:** Characteristics of study population.

	COVID-19 (*n* = 56)	MIS-C (*n* = 65)	Other diseases (*n* = 40)	Convalescent COVID-19 (*n* = 47)
*Age median (years, IQR)*	5.5 (1–17)	6.4 (1–14)	5.8 (1–12)	4.4 years (1–17)
*Male n (%)*	29 (52%)	30 (46%)	22 (55%)	35 (74%)
*RT-PCR positive n (%)*	56% (100%)	0	0	0
*Serology IgG positive n (%)*	0	65 (100%)	5 (13%)	47 (100%)
*Underlying conditions n (%)*	10 (18%)	4 (6%)	22 (55%)	11 (23%)
*Co-existing infections n (%)*	4 (7%)	5 (8%)	NA	6 (13%)
*Median duration since proven or suspected COVID illness or contact (weeks, range)*	NA	3 w (10 d–4 w)	NA	3.2 w (10 d–5 w)
**COVID-19 symptoms *n* (%)**				
*Fever*	47 (84%)	65 (100%)	20 (50%)	17 (36%)
*Gastrointestinal*	20 (36%)	52 (80%)	22 (55%)	15 (32%)
*Respiratory*	15 (27%)	14 (22%)	11 (28%)	16 (34%)
*Mucocutaneous*	0	49 (75%)	5 (13%)	0
*Asymptomatic*	8 (14%)	0	0	30 (64%)
Cardiovascular symptoms/signs *Hypotension* *Shock* *Coronary artery dilatation* *Mycocardial dysfunction*	1 (2%) 1 (2%) 0 0	34 (52%) 21 (32%) 5 (8%) 34 (52%)	9 (23%) NA 0 9 (23%)	0 2 0 0
**Laboratory parameters**				
*CRP* (<*3 mg/L)*	7.2 (<3–181)	101 (3.5–473)	21.3 (<3–78)	5 (<5–181)
*WBC 10^3^ cells/ul* *Geo Mean/Range*	5.319 (3.020–8.390) (*n* = 14)	4.284 (2.77–8.870) (*n* = 21)	8.283 (1.060–29.83)	NA
*Hb (g/dl)* *Geo Mean/Range*	11.23 (8.70–13.09) (*n* = 14)	11.05 (9.20–14.65) (*n* = 21)	9.82 (4.68–15.23)	NA
*Lymphocyte(/mm3)* *(1,500–4,000) median (IQR)*	3,873 (650–12,000)	1,343 (330–6,270)	7,800 (2,400–28,600)	3,890 (650–12,000)
*Neutrophils (/mm3)* *(1,500–7,000) median (IQR)*	3,716 (120–13,160)	11,179 (8,500–15,900)	6,100 (810–9,490)	6,300 (120–13,160)
*Platelets (200–450)* × *10^9^/L median (IQR)*	271 (116–435)	107 (58–255)	290 (100–810)	327 (100–540)
*Sodium (135–145mmol/l) median (IQR)*	137 (135–145)	133 (124–139)	136 (131–148)	138 (135–148)
*Ferritin (ng/ml)* *(7 to 140) median (IQR)*	NA	1,348 (306–5,377)	527 (13–7,200)	NA
Median duration of stay	3.5 (1–9)	5 (3–18)	8 (1–21)	3 (1–10)
*IVIG*	0	44 (68%)	3 (8%)	0
*Steroids*	2 (5%)	47 (72%)	8 (20%)	2 (4%)
*PICU admission*	4 (9%)	34 (52%)	20 (50%)	6 (11%)
*Antibiotics*	13 (30%)	60 (92%)	33 (83%)	16 (31%)
*Tocilizumab (8 mg/kg)*	0	3 (5%)	0	0
**Respiratory support *n* (%)** *Mechanical ventilation* *HHFNC* *Oxygen*	0 2 (5%) 3 (7%)	1(2%) 5 (8%) 9 (14%)	11 (28%) 5 (13%) 1 (3%)	0 2 (4%) 5 (10%)
**Cardiovascular support *n* (%)** *Inotropes* *Fluid Bolus*	0 2 (5%)	34 (52%) 43 (66%)	9 (23%) 6 (15%)	0 2 (4%)

**TABLE 2:** Additional features of children with other diseases.

Total (*n*)	40
Male *n* (%)	22 (55%)
Age (Median, IQR)	5.8 y (1–12 years)
Diagnosis *Dengue fever* *Scrub typhus* *Typhoid* *Acinetobacter sepsis* *Urinary tract infection* *No microorganism isolated*	*n* (%) 3 (8%) 5 (13%) 3 (8%) 2 (5%) 3 (8%) 3 (8%)
Underlying diagnosis *Diabetic ketoacidosis* *Kawasaki’s disease* *Juvenile idiopathic arthritis* *Guillain-Barre syndrome* *Systemic Lupus Erythematosus* *Chronic renal failure* *Hypothyroidism*	*n* (%) 6 (15%) 3 (8%) 1 (3%) 3 (8%) 4 (10%) 3 (8%) 1 (3%)
**Clinical symptoms** *Fever* *Respiratory* *Gastrointestinal* *Mucocutaneous* *Neuromuscular (Headache, abnormal gait, etc.)* *Renal*	20 (50%) 11 (28%) 22 (55%) 5 (13%) 6 (15%) 5 (13%)
Underlying conditions (*n* = 1) *Neurodevelopmental delay*	1

Bold values are subdivision of the clinical characteristics.

For analyses, children were classified into five groups: MIS-C (*n* = 65), acute COVID-19 (*n* = 56), other diseases (*n* = 43), convalescent (*n* = 47) and control (*n* = 21). There is no published evidence concerning these research objectives at the beginning of this study. Therefore, the formal sample size for the study was not calculated and a convenient sample was obtained. Blood was collected in EDTA tubes (BD Biosciences) and heparin tubes and processed within 4 h of collection at the National Institute for Research in Tuberculosis (NIRT), Chennai. Sampling in all children was done prior to receiving any immunomodulatory treatment. To avoid the measurement bias and to increase the precision of the estimates for the accuracy of the assay, the study staff involved in immunological assays were blinded to any clinical data.

### Measurement of matrix metalloproteinases

Circulating plasma levels of MMP-1, MMP-2, MMP-3, MMP-7, MMP-8, MMP-9, MMP-10, MMP-12 and MMP-13 were determined using a multiplex enzyme-linked immunosorbent assay system using the Luminex Magpix Multiplex Assay system; Bio-Rad. MMP level were measured using a commercially available kit (Luminex Human Magnetic Assay kit 8 Plex from R&D Systems). All the samples were tested in duplicates and averages were used for the analysis. The lowest detection limits were as follows: MMP-1, 115.8 pg/ml; MMP-2, 809 pg/ml; MMP-3, 199.2 pg/ml; MMP-7, 27.7 pg/ml; MMP-8, 31.7 pg/ml; MMP-9, 257.5 pg/ml; MMP-10, 78.4 pg/ml; MMP-12, 18.5 pg/ml; MMP-13, 32.9 pg/ml.

### Statistical analysis

Geometric means (GM) were used for measurements of central tendency. Statistically significant differences between MIS-C, acute COVID, children with other diseases, convalescent COVID and control children were analysed using the Kruskal-Wallis test with Dunn’s multiple comparisons. The Mann–Whitney test was used to compare the levels of MMPs between MIS-C children with pediatric intensive care unit (PICU) versus non-PICU admission as well as between COVID-19 children with mild disease versus moderate/severe disease. Multiple linear regression analyses using Spearman rank correlation coefficients were used to determine correlations between variables. *P* ≤ 0.05 was considered statistically significant and all tests were two sided. Analyses were performed using Graph-Pad PRISM Version 9.0 (GraphPad Software, San Diego, CA, USA). CombiROC analysis was performed using the online site http://combiroc.eu. The optimal cut-offs were determined from the best out of the Youden index, distance and absolute difference methods for all possible cut-off points derived from a classifier (i.e., logistic regression). Principle Component Analysis (PCA) was performed to seek linear combinations of the biomarkers that separate out different clusters corresponding to each biomarker that best explain the variance in the data using the R studio.

## Results

### Baseline characteristics of the study cohort

As shown in Table 1, we included 229 children in the study cohort (65 MIS-C, 56 acute COVID-19, 40 other diseases, 47 convalescent COVID-19 and 21 control children). The median age was 5 years (range 1–17 years) and overall, the genders were equally distributed among the groups. All the enrolled acute COVID-19 children were SARS-CoV-2 RT-PCR positive among which 18% children were asymptomatic, 67% presented with mild symptoms, 9% had moderate symptoms and 6% had severe symptoms needing PICU care. Next, all the MIS-C children were seropositive (IgG) among which 52% had severe disease needing PICU care.

Children with other diseases (*n* = 40) include Dengue fever = 3, Typhoid fever = 3, Scrub typhus = 5, other etiology = 8, Diabetic Ketoacidosis = 6, Kawasaki’s Disease = 3, Juvenile idiopathic arthritis (JIA) = 1, Guillain-Barre syndrome (GBS) = 3, Systemic Lupus Erythematosus (SLE) = 4 and chronic renal failure = 3 (Table 2). Seropositive children (*n* = 47) were included as Convalescent COVID-19 controls, some of whom had previous history of symptomatic COVID-19. Control children (*n* = 21) were both SARS-CoV-2 RT-PCR negative and seronegative with a similar median age (6 years, IQR: 1–15 years) and sex (male: 52%, 11/21) (Table 1).

### Heightened plasma levels of matrix metalloproteinases in multisystem inflammatory syndrome in children in the study cohort

**FIGURE 1:**
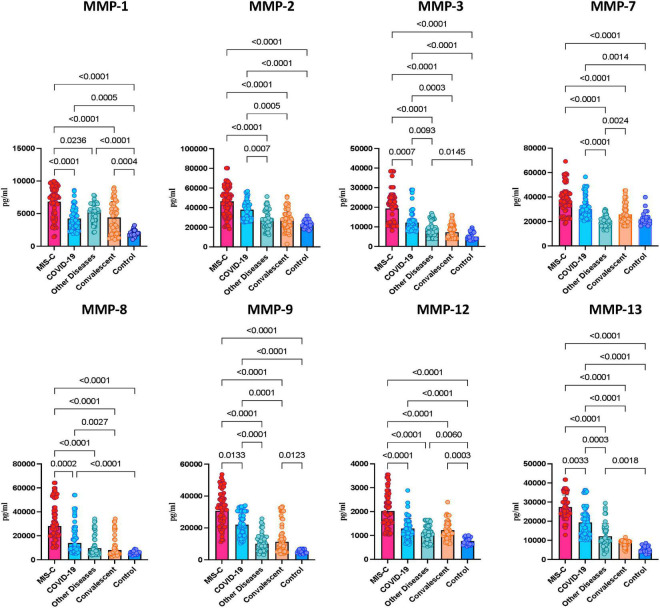
Heightened plasma levels of matrix metalloproteinases (MMPs) in multisystem inflammatory syndrome in children (MIS-C) and acute COVID-19 children. The plasma levels of MMP-1, 2, 3, 7, 8, 9, 12 and 13 were measured in MIS-C (*n* = 65), acute COVID-19 (*n* = 56), children with other diseases (*n* = 40), convalescent COVID-19 (*n* = 47) and control children (*n* = 21). The data are represented as scatter plots with each circle representing a single individual. *p* values were calculated using the Kruskal-Wallis test with Dunn’s *post hoc* for multiple comparisons.

To determine whether MMPs are able to differentiate the different clinical phenotypes of SARS-CoV-2 infection in children, we measured the plasma levels of MMPs-1, 2, 3, 7, 8, 9, 12 and 13. As illustrated in Figure 1, MIS-C children exhibited significantly heightened levels of MMPs-1, 2, 3, 8, 9, 12 and 13 when compared with acute COVID-19 and/or children with other diseases and/or convalescent COVID-19 and/or control groups. Thus, heightened plasma levels of MMPs are associated with MIS-C in children.

### Principle component analysis and receiver operating characteristic analysis of matrix metalloproteinases clearly differentiates multisystem inflammatory syndrome in children from COVID-19 and control groups

**FIGURE 2:**
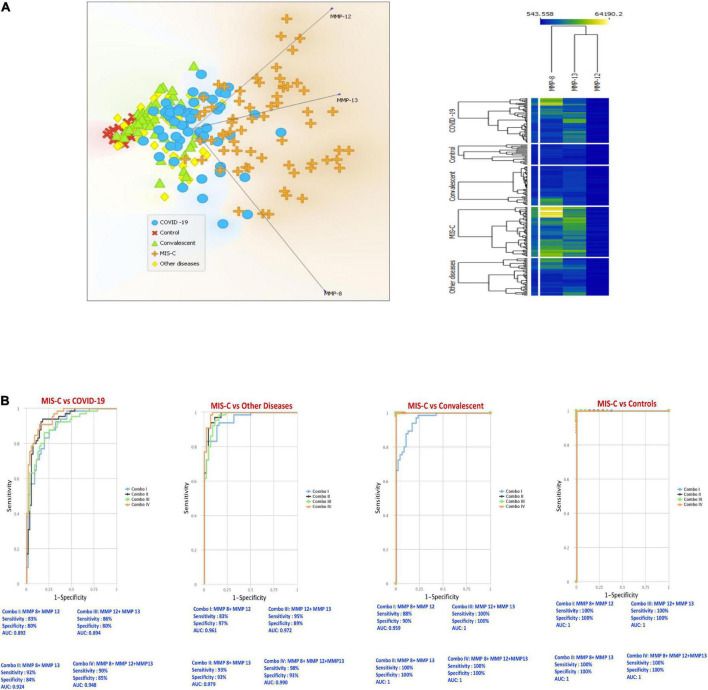
PCA and ROC reveals the trend of matrix metalloproteinases (MMPs) among multisystem inflammatory syndrome in children (MIS-C), COVID-19 and other groups. **(A)** Principal component analysis (PCA) was performed to show the distribution of data from the combination of five groups: MIS-C (brown), acute COVID-19 (blue), children with other diseases (yellow color), convalescent COVID-19 (green) and control (red) children depicted using normalized data from plasma levels of MMP-8, 12 and 13. **(B)** CombiROC analysis was performed to determine the role of MMP 8, 12 and 13 in distinguishing between MIS-C vs. acute COVID-19, MIS-C vs. other diseases, MIS-C vs. convalescent and MIS-C vs. Controls.

To evaluate whether MMPs can help determine the differences between MIS-C and the other groups, we performed PCA (principal component analysis) between the groups on the whole data set and envisaged the clustering pattern of MMPs in above mentioned group of children. We excluded factors with commonalities as low as 0.5 and assessed MMP-8, MMP-12 and MMP-13. As described in Figure 2A, PCA analysis showed that MMPs clusters clearly differentiated MIS-C from acute COVID-19, children with other diseases, convalescent COVID-19 and control group of children.

We performed area under the receiver operating characteristic (ROC) curve (Figure 2B) analysis to assess whether the best possible combination of MMPs is able to discriminate MIS-C from acute COVID-19, children with other diseases, convalescent COVID-19 and controls. A combination of 3 MMPs (MMP-8, MMP-12 and MMP-13) produced an area under the curve (AUC) of 0.89–1, indicating that these MMPs could distinguish MIS-C from acute COVID-19 with 83–92% sensitivity and 80–85% specificity. Next, we performed CombiROC between MIS-C and children with other diseases groups to assess if MMPs could distinguish between them and demonstrated that a combination of 3 MMPs (MMP-8, MMP-12 and MMP-13) produced an area under the curve (AUC) of 0.96–0.99, indicating that these MMPs could distinguish MIS-C from other diseases groups with 83–98% sensitivity and 89–97% specificity. In addition, we also performed CombiROC between MIS-C and convalescent COVID-19 children to assess if MMPs could distinguish between them and demonstrated that a combination of 3 MMPs (MMP-8, MMP-12 and MMP-13) produced an area under the curve (AUC) of 0.95–1, indicating that these MMPs could distinguish MIS-C from convalescent children with 88–100% sensitivity and 90–100% specificity. Finally, we also performed CombiROC between MIS-C and control children to assess if MMPs could distinguish between them and demonstrated that a combination of 3 MMPs (MMP-8, MMP-12, and MMP-13), produced an area under the curve (AUC) of 1, indicating that these MMPs could distinguish MIS-C from other diseases groups with 100% sensitivity and 100% specificity.

### Heightened plasma matrix metalloproteinase levels are associated with disease severity in multisystem inflammatory syndrome in children and COVID-19

**FIGURE 3:**
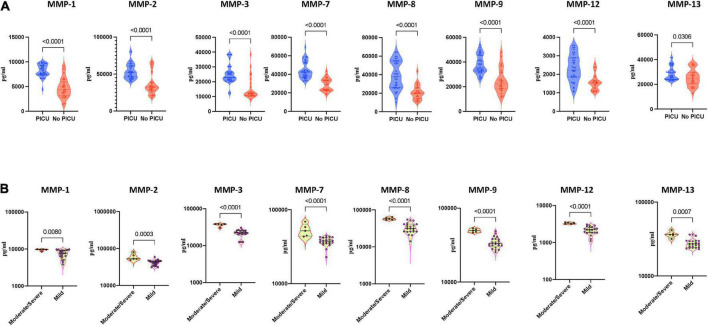
Heightened plasma matrix metalloproteinases (MMP) levels are associated with disease severity in multisystem inflammatory syndrome in children (MIS-C) and COVID-19. **(A)** The plasma levels of MMPs-1, 2, 3, 7, 8, 9, 12 and 13 were measured in MIS-C children with PICU care (*n* = 39) and MIS-C children no PICU care (*n* = 26). **(B)** The plasma levels of MMPs-1, 2, 3, 7, 8, 9, 12 and 13 were measured in COVID-19 children with moderate to severe (*n* = 5) and children with mild (*n* = 22) disease. The data are represented as scatter violin plots with each circle representing a single individual. *P* values were calculated using the Mann–Whitney test with Holm’s correction for multiple comparisons.

To determine whether MMPs could be used to reflect disease severity in MIS-C and acute COVID-19, we compared the plasma levels of MMPs-1, 2, 3, 7, 8, 9, 12 and 13 between MISC children requiring PICU (pediatric intensive care unit) (denoting severe disease) and children who did not need PICU care (denoting mild/moderate disease). As illustrated in Figure 3, MIS-C children with PICU admission exhibited significantly elevated baseline levels of MMPs-1, 2, 3, 7, 8, 9, 12 and 13 compared to children with no PICU care implying that MMPs are associated with disease severity in MIS-C (Figure 3A) Finally, we examined if the MMPs levels may be used to reflect disease severity in acute COVID-19 children. As shown in Figure 3B, moderate/severe COVID-19 children exhibited significantly elevated baseline levels of MMP-1, 2, 3, 7, 8, 9, 12 and 13 compared to mild COVID-19 children, demonstrating that MMPs are associated with disease severity in COVID-19.

### Correlation between matrix metalloproteinase levels and other clinical laboratory parameters

**FIGURE 4:**
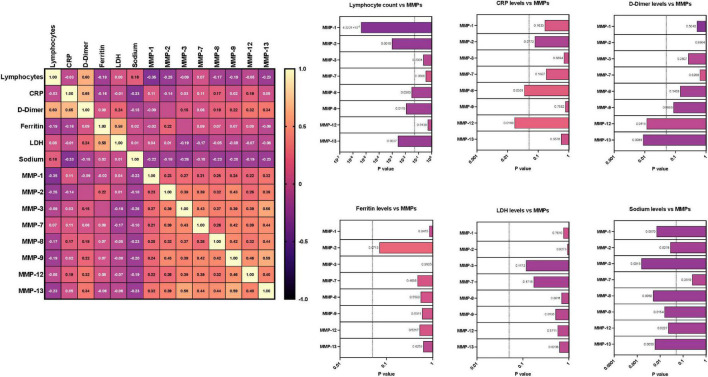
Correlation between matrix metalloproteinases (MMP) levels and other laboratory parameters. Multiparametric matrix correlation plot of MMPs-1, 2, 3, 7, 8, 9, 12 and 13 and laboratory parameters (Lymphocyte, CRP, D-Dimer, Ferritin, LDH, Sodium) with MIS-C and COVID-19. Spearman’s correlation coefficients are visualized by color intensity. *P* values and spearman *r* values are ordered by hierarchical clustering in the study cohort.

We wanted to examine the relationship between MMPs and other laboratory parameters (Lymphocyte, CRP, D-Dimer, Ferritin, LDH, Sodium) in MIS-C, acute, acute COVID-19, children with other diseases, convalescent COVID-19 and control children As illustrated in Figure 4, a multiparametric scatter plot matrix correlation plot exhibited a positive correlation of CRP with MMP 8 and 12, D-Dimer with MMP 12 and 13 and negative correlation of Lymphocytes with MMP 1, 2, 8, 9 and 13, Sodium with MMP 1, 2, 3, 8, 9, 12 and 13 indicating that these clinical laboratory parameters are associated the MIS-C disease status.

## Discussion

COVID-19 in children manifests with a wide range of clinical symptoms ranging from asymptomatic SARS-CoV2 infection; mild, moderate and severe acute COVID-19; convalescent COVID-19 to MIS-C (20). MIS-C is related temporally to SARS-CoV2 infection of unknown pathogenesis that possibly occurs due to the delayed immunological reaction of children to the virus (3, 21). Both MIS-C and COVID-19 can involve multiple organ systems in children. Apart from the respiratory symptoms, children can present with gastro-intestinal, cardiac and muco-cutaneous manifestations as seen in MIS-C and rarely with neurological symptoms (22). The symptoms of MIS-C are most typically similar to Kawasaki disease and/or macrophage activation syndrome (23–26). The immune profile of MIS-C shows changes in innate immune response with elevated pro-inflammatory cytokines, chemokines and enhanced neutrophil and monocyte/macrophage activation, while the changes in adaptive immune response are seen as T cell, B cell and NK cell lymphopenias, enhanced anti-viral antibody responses and elevated circulating autoantibodies (7, 8, 27–30). Our study involves a large number of different COVID-19 groups including acute and convalescent as well as MIS-C and in addition, children with other diseases, who have been very well characterized clinically. As shown in Table 1, we have performed extensive clinical, laboratory and demographic characterization of these children, which adds considerable value to our study findings. In addition, power calculations were carried out to determine the ability of MMPs (MMP8, MMP12, MMP13) to distinguish MIS-C from acute COVID-19 and other tropical diseases. These MMPs differ with an effect of –0.5 to –2.5 and have a power of >90%.

It has been postulated that release of MMPs by neutrophils, macrophages and other cells mediate the extracellular remodeling and tissue pathology in various organ systems that underlie pathology in COVID-19 (31). Since, MIS-C is also a multi-system disease, MMPs may have a major role in the pathogenesis (32). Under steady-state conditions, MMPs are poorly expressed in tissues. However, upon injury, inflammation, extracellular matrix turnover, and repair, their expression is enhanced (33). It has been also reported that overactivation of MMPs may contribute to the dengue pathogenesis and disease severity. In addition, few other studies reported that MMPs might significantly impact the pathogenesis of respiratory viral infections including MIS-C and COVID-19 (33). In addition, studies in adult COVID-19 have reported that circulating levels of MMP-7 are potential biomarkers of disease severity in patients requiring invasive mechanical ventilation (34). Consistent with this hypothesis, our study clearly shows that MIS-C is characterized by elevated levels of MMP-1, 2, 3, 8, 9, 12 and 13. Similarly, acute COVID-19 is also characterized by elevated levels of MMP-3, 7 and 9 in comparison to convalescent COVID-19 and other diseases controls. The PCA analyses substantiates the role of MMPs by displaying the clear distinction of four groups with or without SARS-CoV2 related manifestations. In addition, by using the ROC analysis, we observed that MMPs may serve as significant biomarkers to distinguish MIS-C from acute COVID-19 and other diseases.

Previous studies have examined and identified biomarkers that could differentiate MIS-C from acute and convalescent COVID-19 and healthy control children (17, 35, 36). But very little information is available about the systemic parameters in MIS-C and acute COVID-19 in comparison to children with other diseases, especially those with considerable overlapping clinical presentations and routine laboratory parameters. We fill this knowledge gap by demonstrating major differences in plasma levels of MIS-C from children with other diseases. These diseases include both those of infectious etiology (such as Dengue, Scrub Typhus and Typhoid), which are endemic in India and those of non-infectious etiology (including Kawasaki disease, JIA, SLE), which have a common clinical picture and often confound diagnosis. Our study further highlights the observation that heightened levels of MMPs are an important characteristic of MIS-C and Acute COVID-19 in contrast to children suffering from other illnesses. Our study innovates by including a group of children with other diseases, that despite varied etiologies, have common clinical manifestations that make the final diagnosis of MIS-C very difficult in a country where these diseases are very common.

We also observed that MMPs of children with severe MIS-C and COVID-19 were highly elevated, signifying that MMPs may also be helpful in stratifying the disease severity. The blood sampling in our cohort of children was performed at hospital admission; prior to receiving any immunomodulatory treatment, suggesting that MMPs may be potentially used as baseline biomarkers for predicting disease severity in our population. Finally, we also observed that MMP levels correlate well with laboratory parameters such as CRP, D-Dimer, Ferritin, LDH and Sodium reinforcing the potential contribution of MMPs to pathogenesis of MIS-C and COVID-19. In addition, published studies and meta-analysis have revealed that inflammatory markers, especially WBC, platelets, CRP, ferritin, D-dimer and LDH levels, were correlated with MIS-C and also measurement of these clinical parameters are important for dynamic monitoring of MIS-C and might assist pediatricians to effectively evaluate and manage children and adolescents with MIS-C (37). Findings from this study reveals that MMPs are immune markers of MIS-C and COVID-19 in children.

Our study suffers from the limitation of examining children from a single city and not having any follow up samples to analyse and also other infection group are not homogeneous Despite limitations, our data suggest that MMPs might play a pivotal role in the pathogenesis of MIS-C and COVID-19 in children and may serve as a novel biomarker of both disease severity and biomarker to distinguish MIS-C from other syndromes of overlapping clinical and laboratory presentation. Future studies to corroborate our findings should serve to confirm the role of MMPs as both biomarker and pathogenetic factor of disease in MIS-C and COVID-19 in children.

## Data availability statement

The original contributions presented in this study are included in the article/supplementary material, further inquiries can be directed to the corresponding author.

## Ethics statement

The studies involving human participants were reviewed and approved by NIRT-IEC. Written informed consent to participate in this study was provided by the participants’ legal guardian/next of kin.

## Author contributions

SB and NP designed the study. AN and AR conducted the experiments. AV, AR, and NP acquired the data. NP and KT analyzed the data. SB, UR, TN, and BS contributed the reagents and also revised subsequent drafts of the manuscript. AV, PV, ES, TS, RS, AT, SN, GR, SP, KS, BS, and SH contributed the enrolement of the participants. AV, PV, SN, BS, and SH contributed to acquisition and interpretation of clinical data. SB and NP wrote the manuscript. All authors read and approved the final manuscript.
